# Exploring the Role of Umbilical Cord Blood Leptin and Visfatin in Fetal Growth: Associations with Gestational Diabetes Mellitus and Neonatal Anthropometric Measurements

**DOI:** 10.3390/ijms27198843

**Published:** 2026-10-03

**Authors:** Maria Styliou, Angeliki Tiptiri-Kourpeti, Panagiotis Konstantaras, Panagiotis Tsikouras, Theopi Nalbanti, Nektaria Kritsotaki, Aggeliki Gerente, Konstantinos Nikolettos, Nikolaos Nikolettos

**Affiliations:** 1 Department of Obstetrics and Gynecology, Faculty of Medicine, Democritus University of Thrace, 68100 Alexandroupolis, Greece; mstyliou@gmail.com (M.S.);; 2 Genesis Athens-Thrace Medically Assisted Reproduction Unit, 68100 Alexandroupolis, Greece; 3 Endometriosis Center, University Hospitals of Bristol and Weston NHS Foundation Trust, Trust Headquarters, Bristol BS1 3NU, UK

**Keywords:** leptin, visfatin, adipokines, gestational diabetes mellitus (GDM), fetal growth, umbilical cord blood

## Abstract

Leptin and visfatin are adipokines involved in metabolic regulation and fetal development, but their associations with maternal gestational diabetes mellitus and neonatal growth remain incompletely understood. This study investigated the relationships between umbilical cord blood leptin and visfatin concentrations, maternal GDM status, and neonatal anthropometric characteristics. Umbilical cord blood samples from 88 neonates were analyzed for leptin and visfatin concentrations using ELISA. Statistical analysis was performed using IBM SPSS Statistics v.32. Cord blood leptin concentration was significantly higher in neonates born to mothers with GDM than in those born to mothers without GDM (median [IQR]: 2.22 [1.09] vs. 1.50 [0.77] ng/mL; *p* = 0.002). Visfatin concentration was positively correlated with birth weight (r = 0.359, *p* < 0.01) and head circumference (r = 0.233, *p* = 0.029), and differed significantly across neonatal weight categories (*p* < 0.001). Post hoc analysis showed significant differences in visfatin concentrations between AGA and LGA neonates (adjusted *p* < 0.001) and between SGA and LGA neonates (adjusted *p* = 0.008). In multivariable regression analysis, the models explained 9.3% and 20.5% of the variability in leptin and visfatin concentrations, respectively. Umbilical cord blood leptin and visfatin concentrations were differentially associated with maternal metabolic status and neonatal growth characteristics. GDM status was independently associated with cord blood leptin concentration, whereas neonatal weight category was independently associated with visfatin concentration. These findings suggest that cord blood adipokine concentrations may reflect aspects of the intrauterine metabolic environment and fetal growth; however, their clinical significance and potential predictive value require confirmation in larger longitudinal studies.

## 1. Introduction

Fetal growth is a complex and highly regulated process that depends on the interaction of genetic determinants, maternal nutritional status, placental function, and the intrauterine metabolic environment. Increasing evidence supports the concept of fetal programming, according to which alterations in the intrauterine milieu may induce long-lasting effects on growth trajectories and predispose individuals to obesity, insulin resistance, cardiovascular disease, and other metabolic disorders later in life [1,2]. Consequently, the identification of early biomarkers that reflect fetal growth and metabolic adaptation has become an important area of perinatal research. Evidence also suggests that prenatal therapeutic interventions may influence fetal endocrine and metabolic pathways [3]. Such findings highlight the sensitivity of the fetal metabolic environment to prenatal perturbations. Maternal obesity and diabetes are known to alter the intrauterine metabolic environment and influence fetal hormonal exposure [4]. Adipose tissue secretes adipokines, which participate in the regulation of energy metabolism, insulin sensitivity, inflammation, cellular proliferation, and growth. During pregnancy, adipokines contribute significantly to maternal metabolic adaptations and influence placental function and fetal development [5].

### 1.1. Leptin and Fetal Growth Regulation

Leptin is a 16 kDa peptide hormone predominantly secreted by adipocytes and is primarily involved in the regulation of appetite, energy expenditure, and body weight homeostasis. Beyond its role in postnatal energy regulation, leptin exerts pleiotropic effects during pregnancy and fetal development, participating in placental growth, nutrient transport, angiogenesis, hematopoiesis, neurodevelopment, and the maturation of multiple organ systems [1,6]. During gestation, circulating leptin concentration increases substantially due to both maternal adipose tissue expansion and placental leptin production [6]. Experimental evidence suggests that increased placental leptin expression may contribute to inflammatory processes by reducing the number of myeloid-derived suppressor cells, which are involved in maintaining maternal–fetal immune tolerance [7].

Umbilical cord blood leptin concentration has been proposed as a reliable biomarker of fetal adiposity and intrauterine growth. Numerous studies have demonstrated significant positive associations between cord blood leptin levels and neonatal anthropometric measurements [2,8,9,10]. Elevated leptin concentration has consistently been observed in large-for-gestational-age (LGA) infants, whereas lower concentrations are frequently reported in small-for-gestational-age (SGA) infants [10,11,12]. In a prospective study, leptin concentrations were significantly higher in macrosomic and LGA newborns [13]. Lower methylation levels of the leptin gene promoter in cord blood have been associated with macrosomia and elevated cord blood leptin concentrations, suggesting that alterations in leptin gene regulation may participate in excessive fetal growth even in pregnancies not complicated by gestational diabetes [14]. Interestingly, higher leptin concentrations have been observed in newborns delivered vaginally compared with those delivered by cesarean section, suggesting that labor-associated physiological stress and inflammatory activation may modulate fetal leptin secretion [15]. Significant positive correlations between leptin concentrations and neonatal weight have been observed across diverse populations and clinical settings [6,8,16,17]. In a large consecutive birth cohort, cord blood leptin was significantly associated with gestational age, birth weight, maternal body mass index, infant sex, and plurality [18]. Recent evidence has demonstrated significantly higher cord blood leptin concentrations in neonates born to mothers with GDM compared with controls, regardless of geographical region, analytical methodology, or biological sample type [19]. Pregnancies complicated by fetal growth restriction (FGR) demonstrate significantly reduced umbilical cord blood leptin concentrations [16,20] and disturbances in angiogenic and endothelial pathways compared with uncomplicated pregnancies [21].

Conversely, maternal diabetes and gestational diabetes mellitus are associated with elevated cord blood leptin concentrations, increased birth weight, and greater neonatal adiposity [11,22,23]. Higher cord blood leptin concentrations have been associated with slower weight gain, lower increase in adiposity during early infancy and neurodevelopmental disorders [24,25].

### 1.2. Visfatin and Intrauterine Metabolic Regulation

Visfatin, also known as nicotinamide phosphoribosyltransferase (NAMPT) or pre-B-cell colony-enhancing factor, is another adipokine that has emerged as a potential regulator of metabolic homeostasis during pregnancy. Visfatin is predominantly expressed in visceral adipose tissue but is also produced by placental tissue and fetal membranes. It has insulin-mimetic properties and participates in glucose metabolism, inflammatory responses, cellular differentiation, and energy regulation [5].

Compared with leptin, the physiological role of visfatin during fetal development remains incompletely understood and the available evidence is relatively inconsistent. Higher cord blood visfatin concentrations have been reported in SGA infants compared with AGA and LGA neonates [26]. Similarly, cord blood visfatin concentrations differed significantly among neonatal weight groups and appeared to be associated with fetal growth patterns [10]. In contrast, other investigators found no significant differences in cord blood visfatin concentrations among SGA, AGA, and LGA infants [27].

Nevertheless, maternal metabolic status appears to substantially influence fetal visfatin concentrations. Maternal obesity has been associated with increased visfatin concentrations in both maternal and cord blood [5], while pregnancies complicated by gestational diabetes mellitus exhibit significantly elevated visfatin levels and increased rates of adverse neonatal outcomes [23,28]. Umbilical cord visfatin concentrations have been reported to be significantly higher in newborns of mothers with obesity, while maternal visfatin levels were negatively associated with neonatal anthropometric measurements. Moreover, cord blood visfatin concentrations correlated positively with maternal body weight and body mass index, suggesting that maternal adiposity may influence fetal visfatin exposure and subsequent growth patterns [29]. Maternal serum visfatin levels during early pregnancy have been identified as negative predictors of birth weight, suggesting that maternal adipose tissue and its endocrine activity may contribute significantly to the metabolic environment that shapes fetal growth [30]. Preeclampsia also appears to alter visfatin metabolism [31].

### 1.3. Adipokines as Biomarkers of Neonatal Anthropometric Characteristics: The Role of Maternal Diabetes Mellitus

Anthropometric measurements at birth, including birth weight, birth length, head circumference, and ponderal index, are widely accepted indicators of intrauterine growth and fetal nutritional status. Maternal environmental exposures, including tobacco exposure during pregnancy, may also influence fetal endocrine programming and adipokine profiles [32,33]. Lower fetal adiponectin-to-leptin ratios have been associated with higher birth weight, greater neonatal anthropometric indices, and early alterations in cardiac structure during infancy [34]. Higher leptin levels have been associated with longer gestation, increased birth weight z-scores, maternal overweight and obesity, gestational diabetes, pregestational diabetes, and female sex, whereas lower concentrations have been observed among male infants and preterm neonates [35]. Among pregnancies complicated by GDM, cord blood leptin concentrations have been shown to correlate positively with neonatal size [36]. Exposure to pregestational type 2 diabetes has been associated with increased cord blood leptin, insulin, and glucose concentrations, together with higher birth weights, whereas gestational diabetes appears to exert less pronounced effects when adequately managed during pregnancy [37].

Despite increasing evidence linking cord blood leptin and visfatin concentrations with maternal metabolic status and fetal growth, findings remain inconsistent. Moreover, the independent contribution of maternal GDM and neonatal anthropometric characteristics to cord blood adipokine concentrations remains incompletely characterized. Therefore, the present study aimed to investigate umbilical cord blood leptin and visfatin concentrations and their associations with maternal GDM status and neonatal anthropometric characteristics.

## 2. Results

A total of 88 pregnant women and their neonates were included in the analysis. Neonates’ measurements and mothers’ anthropometric characteristics and social habits are summarized in Table 1 and Table 2, respectively. The mean birth weight was 3136.59 ± 413.09 g and when corrected for gestational age this presented the 32nd and 40th percentile for male and female neonates, respectively. A total of 37.5% were male and 44 infants were AGA (50%), 14 were SGA (15.9%), and 30 were LGA (34.1%). The majority (70.5%) were delivered by cesarean section. Notably, 18.2% were admitted to the NICU; however, only 8% required ongoing care, following the initial clinical evaluation. The data for cases in which a deterioration in Apgar score was observed are presented in Supplementary Table S1.

The mothers had a mean age of 29.88 ± 5.74 years. The mean BMI at 3rd trimester assessment was 30.31 ± 5.64 kg/m^2^ and mean maternal weight was 81.13 ± 15.23 kg. Mean gestational weight change was 12.67 ± 7.20 kg; two women experienced a net weight loss during pregnancy. Overall, 44.3% of women were experiencing their first pregnancy, while 3.4% had more than two previous pregnancies. A total of 17% of pregnant women were diagnosed with gestational diabetes mellitus, including cases with pre-existing insulin resistance. A total of 11.4% of the enrolled women received pharmacological treatment, whereas the remaining participants adapted lifestyle modifications. A total of 18.2% of expectant mothers were smoking during pregnancy and 1.1% reported alcohol consumption.

Among all enrolled participants, the median umbilical cord blood leptin concentration was 2.20 [1.02] ng/mL, while visfatin concentration was 6.70 [3.48] ng/mL, reported as median [IQR] (Table 3). The raw absorbance measurements and corresponding standard curves used for the ELISA analyses are provided in Supplementary Figure S1. Spearman correlation analysis was performed to examine the relationships between cord blood adipokine concentrations and maternal and neonatal characteristics. As summarized in Table 4, visfatin showed a significant positive association with neonatal birth weight (r = 0.359, *p* < 0.01), indicating that higher cord blood visfatin concentration was associated with greater neonatal birth weight. Visfatin was also positively associated with neonatal head circumference (r = 0.233, *p* = 0.029).

**Figure 1:**
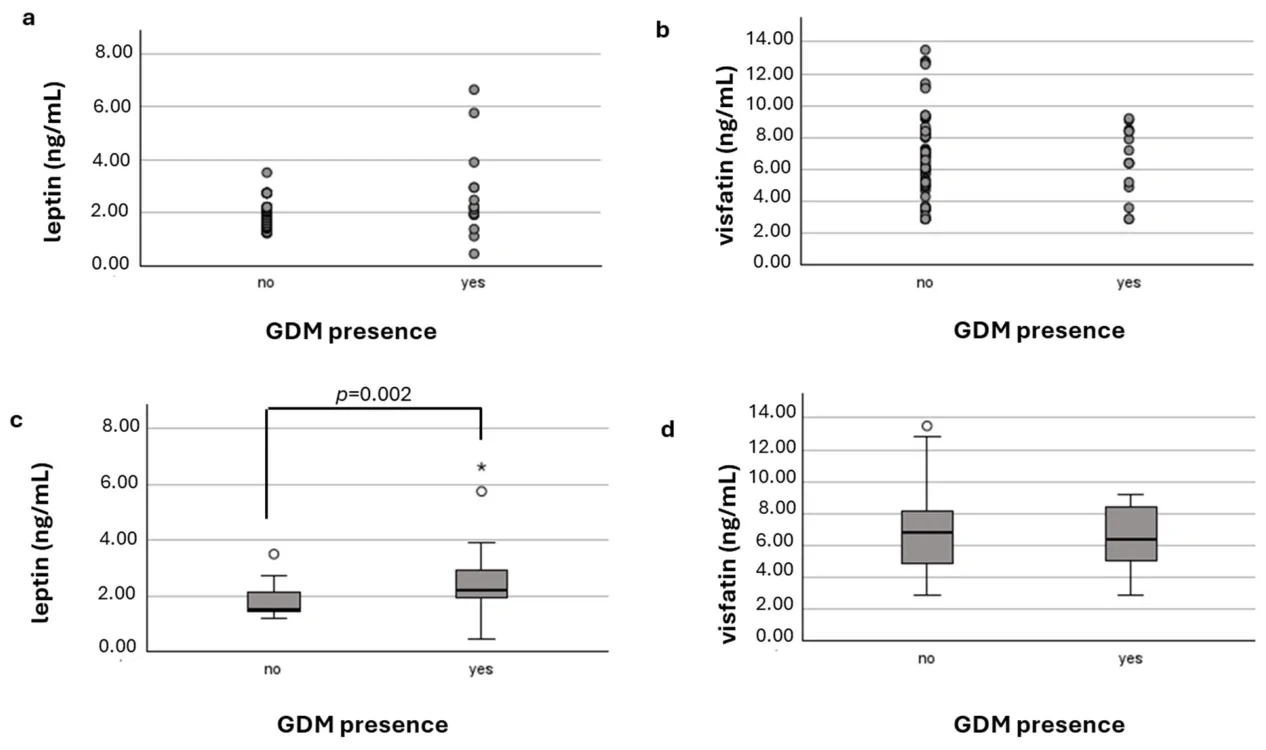
Umbilical cord blood leptin and visfatin concentrations according to gestational diabetes mellitus (GDM) status. The upper panels show the distribution of individual umbilical cord blood leptin (**a**) and visfatin (**b**) concentrations in neonates born to mothers with or without GDM. The lower panels present corresponding box-and-whisker plots for leptin (**c**) and visfatin (**d**) concentrations according to GDM status. In the box plots, the horizontal line within each box represents the median, the box represents the interquartile range (IQR), and the whiskers extend to the most extreme data points within 1.5 × IQR. Individual points beyond the whiskers represent outlying observations. Circles indicate outliers, while asterisks indicate extreme outliers. Concentrations are expressed in ng/mL. Statistical significance was assessed using the Mann–Whitney U test, with *p* < 0.05 considered statistically significant. GDM, gestational diabetes mellitus; IQR, interquartile range.

**Figure 2:**
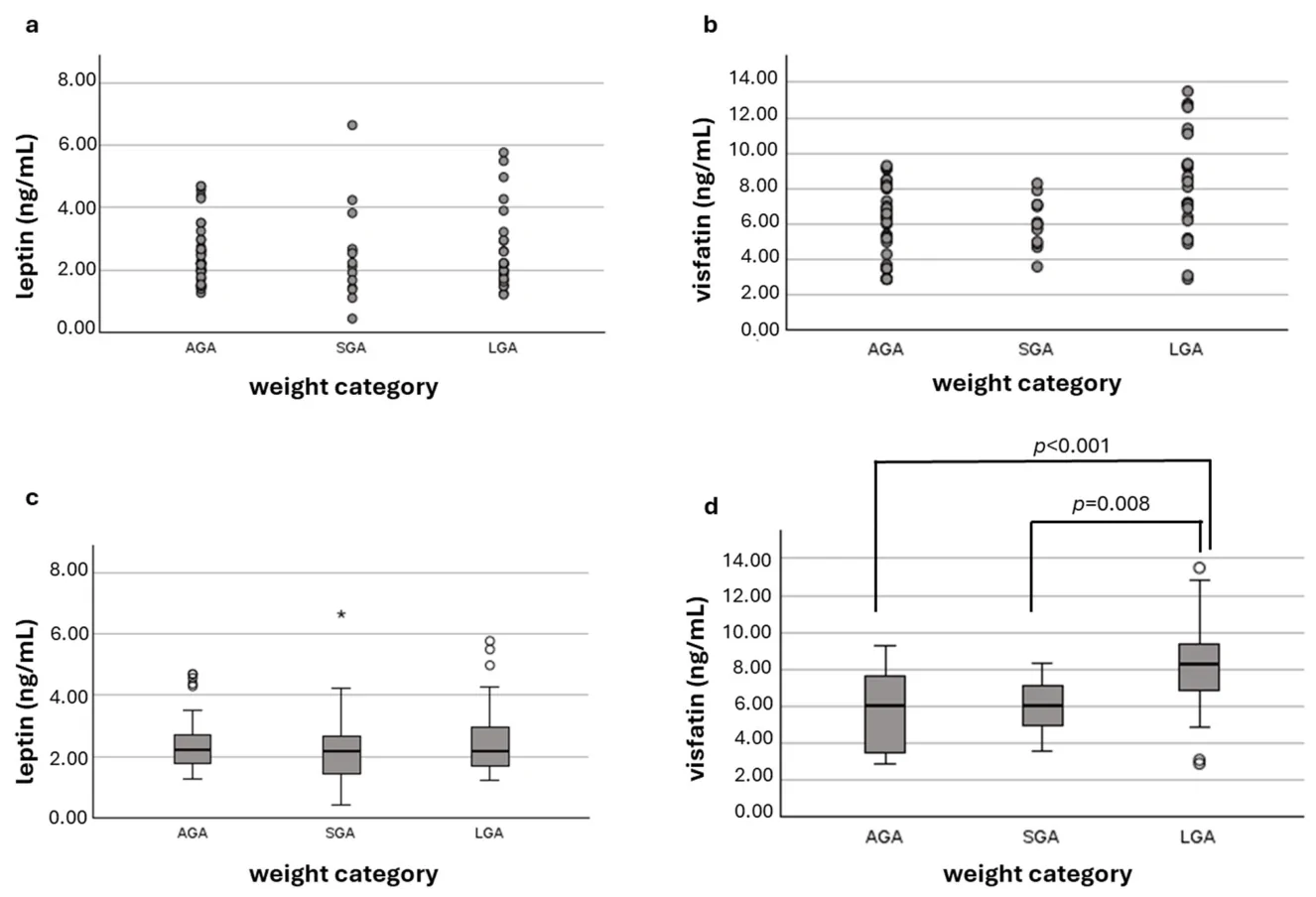
Distribution of umbilical cord blood leptin and visfatin concentrations according to neonatal birth-weight category. Individual data points (upper panels) and box-and-whisker plots (lower panels) showing leptin (**a**,**c**) and visfatin (**b**,**d**) concentrations in neonates classified as appropriate for gestational age (AGA), small for gestational age (SGA), or large for gestational age (LGA). In the box plots, the horizontal line represents the median, the box represents the interquartile range (IQR), and the whiskers extend to the most extreme data points within 1.5 × IQR. Circles indicate outliers, while asterisks indicate extreme outliers. Concentrations are expressed in ng/mL. Statistical significance was assessed using the Kruskal–Wallis test, with *p* < 0.05 considered statistically significant.

The association of cord blood leptin and visfatin concentration with GDM presence and neonatal weight category (SGA, AGA, LGA) is presented in Table 5. Leptin concentration differed markedly according to maternal GDM status, with significantly higher concentrations observed among neonates born to mothers with GDM compared with those born to mothers without GDM (U = 271.00, Z = −3.068, *p* = 0.002). The median [IQR] leptin concentration was 2.22 [1.09] ng/mL in the GDM group, compared to 1.50 [0.77] ng/mL in the non-GDM group (Figure 1). When neonates were categorized according to birth weight status, visfatin concentration differed across the SGA, AGA, and LGA groups (*p* < 0.001; Table 5) (Figure 2). Post hoc pairwise comparisons with Bonferroni correction indicated that visfatin differed significantly between AGA and LGA neonates (adjusted *p* < 0.001) and between SGA and LGA neonates (adjusted *p* = 0.008).

Because leptin and visfatin concentrations exhibited non-normal distributions, their natural logarithmic-transformed values were used as dependent variables in the multivariable regression analyses. The assumptions of linear regression were evaluated before interpretation of the models. Normality of residuals was assessed using normal Q–Q plots, and the residuals were found to be approximately normally distributed. Independence of residuals was confirmed using the Durbin–Watson statistic.

Variables showing an association with leptin in the univariate analyses, including GDM status and mother’s weight gain, were entered into the multivariable model. As presented in Table 6, the multivariable regression model explained 9.3% of the variability leptin concentration (*p* = 0.016). For regression analyses, GDM status was coded as 1 = no GDM and 2 = GDM. GDM status was independently associated with leptin concentrations, with higher log-transformed leptin concentrations observed in neonates of mothers with GDM compared with those without GDM (*p* = 0.017). A separate multivariable model was performed to evaluate the association between statistically significant parameters (neonatal weight category, neonatal birth weight, head circumference and neonatal length) and visfatin concentration. The overall regression model was statistically significant (*p* < 0.001), explaining 20.5% of the variability in visfatin concentration. Neonatal weight category was identified as a significant independent predictor of visfatin concentration (*p* = 0.002). No problematic multicollinearity was identified among the predictors, with tolerance values ranging from 0.278 to 0.742 and VIF values ranging from 1.348 to 3.598. Expanded multivariable models including additional covariates were also evaluated for leptin and visfatin. The inclusion of these variables did not alter the main findings, with GDM status and neonatal weight category remaining the only independent predictors of leptin and visfatin concentrations, respectively.

## 3. Discussion

The present study investigated the associations between umbilical cord blood concentrations of leptin and visfatin with neonatal anthropometric characteristics and maternal gestational diabetes mellitus. The principal findings were that cord blood leptin concentrations were significantly higher in neonates born to mothers with GDM, while visfatin concentrations were positively associated with neonatal birth weight and head circumference. Furthermore, visfatin differed according to neonatal weight category, demonstrating a particularly strong association with LGA status. Multivariable regression analysis identified GDM as an independent factor associated with cord blood leptin concentration and neonatal weight category as an independent factor associated with visfatin concentration.

One of the most important findings of this study was the significantly elevated cord blood leptin concentrations observed in neonates born to mothers with GDM compared with those born to non-diabetic mothers. This association remained significant after adjustment for mothers’ weight gain, indicating that GDM status remained independently associated with cord blood leptin concentrations in our cohort. These findings are consistent with the growing body of evidence demonstrating increased cord blood leptin concentrations in pregnancies complicated by GDM [19,22,23].

The observed elevation of leptin in GDM pregnancies may be explained by several biological mechanisms. Maternal hyperglycemia leads to increased placental glucose transfer, resulting in fetal hyperinsulinemia and enhanced adipose tissue deposition. Since leptin is predominantly produced by adipocytes, increased fetal fat mass is expected to elevate circulating leptin concentrations. In addition, the placenta itself produces leptin, and placental leptin expression has been shown to increase in diabetic pregnancies, potentially contributing to higher fetal leptin exposure [1,6]. The persistence of GDM as an independent factor associated with cord blood leptin concentration suggests that the observed association between maternal metabolic status and fetal leptin concentrations may not be explained solely by neonatal size.

The linear regression model explained 9.3% of the variability in leptin concentrations, indicating a relatively limited explanatory power. The inclusion of additional predictors (Apgar score, smoking habit, alcohol consumption and neonatal anthropometric characteristics) in the expanded model did not materially improve the proportion of explained variance, suggesting that the variables examined accounted for only a modest proportion of the observed variability in leptin concentrations. Previous studies have identified maternal body mass index, gestational age, infant sex, placental function, and genetic or epigenetic factors as significant determinants of cord blood leptin concentrations [14,18]. Therefore, the unexplained variance in our model likely reflects the multifactorial regulation of fetal leptin production.

Leptin concentrations did not differ across neonatal weight categories However, lower leptin concentrations in SGA neonates have been consistently reported in the literature and are thought to result from reduced fetal fat mass and impaired placental nutrient transfer [16,20]. Conversely, elevated leptin concentrations in LGA infants have been associated with increased fetal adipose tissue accumulation and fetal overgrowth [10,11]. The lack of an independent association between neonatal weight category and leptin may also reflect the relatively small sample size and the limited number of SGA and LGA infants included in the study.

In contrast to leptin, visfatin concentrations demonstrated significant positive correlations with neonatal birth weight and head circumference. These findings suggest that visfatin may be associated with fetal growth and somatic development. The positive association with birth weight is consistent with studies reporting higher visfatin concentrations in neonates with greater birth weight and altered fetal growth patterns [10]. The correlation with head circumference is particularly noteworthy, as it may indicate a relationship between visfatin and overall fetal growth, including cranial development.

Visfatin possesses insulin-mimetic properties and participates in glucose metabolism, inflammatory responses, and energy regulation [5]. Therefore, elevated visfatin concentrations in larger neonates may reflect increased fetal metabolic activity and enhanced nutrient utilization. The placenta is also a source of visfatin, and placental visfatin expression may increase in response to maternal metabolic disturbances, further influencing fetal visfatin exposure.

Visfatin concentration differed significantly among neonatal weight categories, with post hoc analyses revealing higher concentrations in LGA neonates compared to both AGA and SGA infants. This finding is consistent with an association between cord blood visfatin concentrations and neonatal growth pattern, particularly LGA status. Similar results have been reported by Pekal et al. (2022), who observed significant differences in visfatin concentration according to neonatal weight category [10].

However, the literature regarding visfatin and fetal growth remains controversial. Yalinbas et al. (2019) reported higher visfatin concentration in SGA infants, suggesting a compensatory metabolic response to adverse intrauterine conditions [26], whereas Estrada-Zúñiga et al. (2018) found no significant differences among SGA, AGA, and LGA neonates [27]. The discrepancies among studies may be attributed to differences in study populations, maternal metabolic characteristics, gestational age, sample size, and analytical methodologies.

In our study, neonatal weight category remained an independent predictor of visfatin concentration, with the multivariable regression model explaining 20.5% of the observed variability. This finding strengthens the evidence that visfatin is closely associated with fetal growth patterns, particularly LGA status. The independent association suggests that visfatin concentrations may be related to fetal growth characteristics independently of GDM status in our cohort.

A negative correlation was observed between maternal age and gestational age, indicating that older maternal age was associated with shorter gestation in our cohort. Advanced maternal age has been associated with an increased risk of obstetric complications, including preterm birth, placental dysfunction, and hypertensive disorders of pregnancy. Although this association was not directly related to adipokine concentrations in our study, it underscores the complex interplay between maternal characteristics and fetal development.

The findings of this study support the concept of fetal programming, whereby alterations in the intrauterine metabolic environment influence fetal endocrine regulation and growth trajectories. Elevated leptin concentrations in GDM pregnancies and increased visfatin concentrations in LGA neonates suggest that maternal metabolic disturbances may modify fetal adipokine exposure, potentially predisposing offspring to future metabolic disorders. Previous studies have linked altered cord blood adipokine profiles with obesity, insulin resistance, cardiovascular disease, and metabolic syndrome later in life [2,34]. The identification of adipokine alterations at birth may facilitate the early recognition of newborns at increased risk for adverse metabolic outcomes.

### Strengths and Limitations

The present study has several strengths, including the simultaneous evaluation of two biologically relevant adipokines, the use of standardized neonatal anthropometric measurements, and the application of multivariable regression analyses to identify independent predictors of adipokine concentrations. In addition, the study included detailed maternal anthropometric and clinical data, allowing for a comprehensive assessment of maternal factors influencing fetal adipokine exposure.

However, certain limitations should be acknowledged. The sample size was relatively small, particularly for subgroup analyses involving neonates’ weight category, and GDM cases, which may have limited the statistical power to detect weaker associations. In particular, the small number of pharmacologically treated GDM cases precluded a robust comparison between pharmacological and lifestyle-based management and their potential associations with cord blood leptin and visfatin concentrations. Accordingly, the observed differences in cord blood adipokine concentrations should be interpreted as associations with maternal GDM status rather than as evidence of a causal effect of GDM or its treatment.

A limitation of the present study is the variability observed in a proportion of duplicate measurements, although observed among samples with very low absorbance values. Because the original samples were no longer available, repeat measurements could not be performed. Therefore, the original measurements were retained for analysis, and this analytical variability should be considered when interpreting the leptin concentrations and related statistical associations. Therefore, this analytical variability should be considered when interpreting the leptin concentrations and the related statistical associations.

The cross-sectional design precludes the establishment of causal relationships between maternal metabolic status, adipokine concentrations, and neonatal anthropometric outcomes. Furthermore, maternal serum leptin and visfatin concentrations were not measured, preventing direct comparison between maternal and fetal adipokine profiles. Finally, the study did not assess mothers’ BMI pre-gestational, neonatal body composition or long-term metabolic outcomes, which would provide additional insight into the clinical significance of altered adipokine exposure.

## 4. Materials and Methods

### 4.1. Patient Selection and Data Classification

A total of 88 infants born at the Department of Gynecology and Obstetrics, Democritus University Faculty of Medicine, between July 2024 and November 2025 were enrolled in this study. The study protocol was approved by Ethics Committee of the Faculty of Medicine, Democritus University of Thrace (EΣ4, 60201/7 December 2023; Approval Date: 15 February 2024) and written informed consent was obtained from all participants involved in the study. This research received no external funding.

Gestational age was calculated based on the first day of the last menstrual period. The first-trimester ultrasound measurements were reviewed, and no significant differences were identified. All infants included were term neonates and had a gestational age >36 weeks and type of birth (vaginal birth or cesarean section) was documented. Neonates were classified according to their birth-weight percentile using WHO Multicentre Growth Reference Study charts from birth to 24 months old. Neonates with a birth weight below the 10th percentile were considered small for gestational age (SGA), those with a birth weight between the 10th and the 90th percentiles were considered appropriate for gestational age (AGA), and those with a birth weight above the 90th percentile were considered large for gestational age (LGA) [38]. Sex was determined, and the infant’s weight, length and head circumference were measured. Body weight was measured using electronic balances sensitive to the nearest 10 g. Body length was measured using a standardized length board with the infant in the supine position, with one person holding the baby’s head against the fixed end of a measuring set and a second person placing the baby’s foot onto the moving part of the measuring set followed by the full extension of the legs, as described by Yalinbas et al., 2019 [26]. Head circumference was measured over the most prominent part on the back of the head using a nonflexible paper measuring tape. Additionally, the Apgar score was determined at 1, 5 and 10 min postnatal during the neonate’s resuscitation, and the possible admission or brief stay of each neonate in the Neonatal Intensive Care Unit was recorded.

Mother anthropometric characteristics and social habits were recorded. Parity was calculated based strictly on past, completed pregnancies, explicitly excluding the current index pregnancy. Maternal body weight was measured during the 3rd trimester using an electronic balance sensitive to the nearest 10 g. Maternal pre-pregnancy weight was recorded from the medical records or, when unavailable, obtained by self-report. Gestational weight change was additionally recorded. Body mass index (BMI) was calculated at the 3rd trimester assessment as weight in kilograms divided by the square of height in meters (kg/m^2^). All women categorized as smokers were daily users. To evaluate the intensity of tobacco consumption, participants were categorized into distinct smoking tiers based on their self-reported daily cigarette consumption (cigarettes per day). Consistent with previously described epidemiological literature [39,40], smoking intensity was operationalized using five categories: (a) non-smokers (no cigarette use for more than 1 year); (b) light—individuals who reported consuming between 1 and 10 CPD; (c) moderate—individuals who reported consuming between 11 and 19 CPD; (d) heavy smoking—individuals who reported consuming 20 or more CPD; and (e) individuals that preferred other smoking alternatives (vapes and heated tobacco products). No further sub-classification or stratification was conducted based on the frequency, intensity, or behavioral patterns of alternative product utilization, mainly due to an insufficient sample size within these sub-cohorts. Alcohol intake within the study group classified participants into two mutually exclusive groups based on their self-reported drinking status. Individuals reporting active alcohol intake during pregnancy were categorized as consumers, while those reporting complete abstinence were classified as non-consumers. No further stratification was performed regarding the frequency, volume, or specific type of alcohol consumed. Lastly, gestational diabetes mellitus (GDM) was clinically diagnosed based on impaired glucose tolerance recognized during the current pregnancy and was evaluated using two separate binary (yes/no) subgroups. GDM was diagnosed according to the IADPSG criteria using a 75 g oral glucose tolerance test (OGTT) [41]. A history of insulin resistance predating pregnancy was recorded separately as a maternal clinical characteristic. GDM was diagnosed when one or more of the following venous plasma glucose values were met: fasting plasma glucose 92–125 mg/dL, 1 h plasma glucose ≥180 mg/dL, or 2 h plasma glucose 153–199 mg/dL following the 75 g glucose load. Women meeting the criteria for diabetes mellitus in pregnancy (fasting plasma glucose ≥ 126 mg/dL or 2 h plasma glucose ≥ 200 mg/dL) were considered separately. A subset of women with GDM had a documented history suggestive of insulin resistance predating pregnancy. Therapeutic management of GDM was based on clinical severity, with some women requiring pharmacological treatment to achieve adequate glycemic control, while others were managed with dietary modifications alone.

### 4.2. Sample Collection and Plasma Analysis

**Figure 3:**
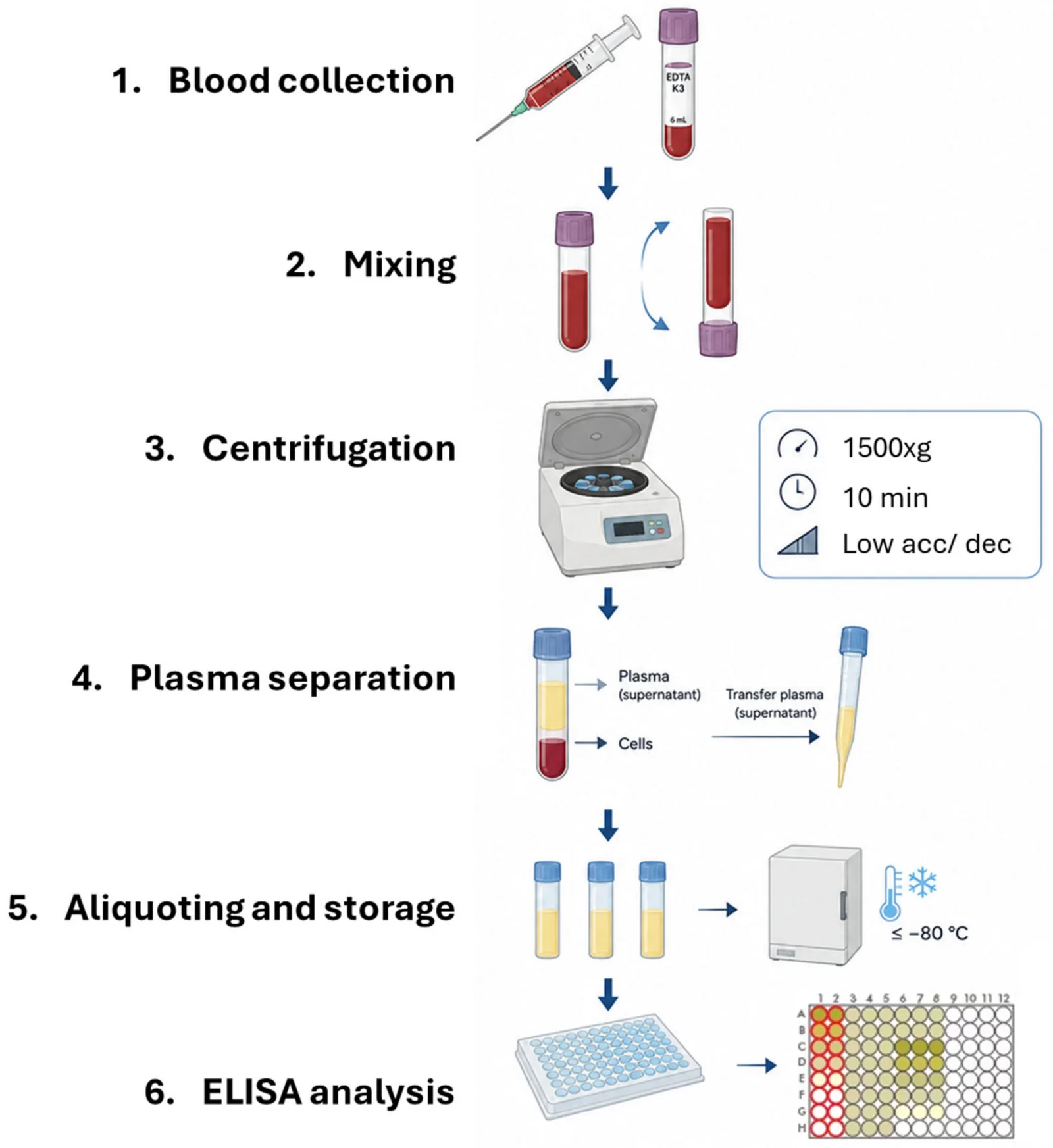
Plasma collection, processing, storage, and ELISA workflow. Umbilical cord blood was collected in K2-EDTA tubes, gently mixed, and centrifuged to separate plasma. Plasma was aliquoted into cryovials and stored at −80 °C until analysis by ELISA.

Umbilical cord blood samples were collected at the time of delivery into vacutainers K2 EDTA, 6 mL (BD Vacutainer Plus, 3.6 mg EDTA K2 Tubes, Becton, Dickinson and Company, Franklin Lakes, NJ, USA). To minimize hemolysis, venipuncture was performed using a standard syringe, avoiding the use of butterfly needles whenever possible. Blood samples were centrifuged within 10 min of collection (or within 30 min when maintained on ice) at 1500× *g* for 10 min using low acceleration and deceleration settings to minimize cellular disruption. Plasma was carefully aspirated from the supernatant. If residual cellular debris was observed, samples were subjected to an additional centrifugation step at 1500× *g* for 5 min. Samples exhibiting visible hemolysis were excluded from further analysis. Plasma was aliquoted into polypropylene cryovials in volumes of at least 0.5 mL to avoid repeated freeze–thaw cycles and was stored immediately at ≤−80 °C for subsequent enzyme-linked immunosorbent assay (ELISA) measurements. A schematic overview of the experimental workflow is presented in Figure 3. For leptin quantification, plasma samples were diluted 1:4 prior to analysis, according to the assay requirements. Human leptin and human visfatin C termina were quantified in duplicate using a custom ELISA kit (Leptin Cat. No. EH0216, Visfatin Cat. No. EH4159, Wuhan Fine Biotech Co., Ltd., Wuhan, China) according to manufacturer’s instructions. The assay detects minimum concentrations of 31.25 pg/mL for leptin and 0.313 ng/mL for visfatin. The optical density of the reaction products was measured using an ELISA plate reader (EnSpire Multimode Plate Reader, Turku, Finland). Absorbance values were corrected by subtraction of the blank absorbance prior to calibration and concentration determination. Data were analyzed with Sigma Plot Software v.11 (Systat Software GmbH, Erkrath, Germany). A four-parameter logistic (4PL) regression model was fitted to the blank-subtracted absorbance values of the standards, and the resulting calibration curve was used to determine the adipokine concentrations of the unknown samples.

### 4.3. Statistical Analysis

Statistical analyses were performed using IBM SPSS Statistics for Windows, Version 32.0 (IBM Corp., Armonk, NY, USA). Data are expressed as mean ± standard deviation (SD) for continuous variables with normal distribution and median (IQR) for continuous variables with non-normal distribution, while categorical variables are presented as frequencies and percentages. The distribution of continuous variables was assessed using the Shapiro–Wilk test for normality. Since the examined hormones did not follow a normal distribution, non-parametric statistical methods were applied. Associations between continuous variables were evaluated using Spearman’s rank correlation coefficient. Differences in continuous variables between two independent categorical groups were assessed using the Mann–Whitney U test, whereas comparisons among more than two categorical groups were performed using the Kruskal–Wallis test. The Hodges–Lehmann estimator was used to estimate the location shift between groups, with corresponding 95% confidence intervals. Variables showing statistically significant associations in univariate analyses were subsequently included in multivariable regression models to identify independent predictors of hormone levels. Regression analyses were performed after assessing the relevant model assumptions, including linearity, homoscedasticity, independence of residuals, and absence of multicollinearity among predictors. Regression coefficients (β), corresponding *p* values, and the coefficient of determination (R^2^) were reported. For regression analyses, GDM status was coded as 1 = no GDM and 2 = GDM. A two-tailed *p* value < 0.05 was considered statistically significant.

Generative artificial intelligence has been used in this paper to generate the graphics of Figure 3.

## 5. Conclusions

This study identified differential associations between umbilical cord blood leptin and visfatin concentrations, maternal GDM status, and neonatal growth characteristics. Cord blood leptin concentrations were significantly higher in neonates born to mothers with GDM and remained independently associated with GDM status after multivariable adjustment. In contrast, visfatin concentrations were positively associated with neonatal birth weight and head circumference and differed significantly according to neonatal weight category, with higher concentrations observed in LGA neonates than in AGA and SGA neonates. Neonatal weight category remained independently associated with visfatin concentration in the multivariable model. These findings suggest that leptin and visfatin may reflect distinct aspects of the maternal–fetal metabolic environment and fetal growth. Nevertheless, the observational design, relatively small sample size, and absence of longitudinal follow-up preclude conclusions regarding causality or long-term clinical significance. Larger prospective studies incorporating maternal and neonatal metabolic profiles and long-term follow-up are warranted to clarify the biological significance and potential clinical relevance of cord blood leptin and visfatin.

## Figures and Tables

**Table 1:** Neonatal anthropometric and clinical characteristics of the study population. Data are presented as mean ± standard deviation for continuous variables and frequency (*n*) and percentage (%) for categorical variables. SGA, small for gestational age; AGA, appropriate for gestational age; LGA, large for gestational age; NICU, neonatal intensive care unit.

		Minimum	Maximum	Mean	Standard Deviation
Head circumference (cm)		29	37	33.81	1.52
Body length (cm)		42	56	49.89	2.16
Gestational age (weeks)		36.86	40.29	38.63	0.87
Birth weight (g)		2180	3985	3136.59	413.09
			Frequency (*n*)	Percent (%)	
Weight category	SGA		14	15.9	
	AGA		44	50	
	LGA		30	34.1	
Birth type	Vaginal		26	29.5	
	Cesarean		62	70.5	
Gender	Male		33	37.5	
	Female		55	62.5	
Apgar score—1 min	0–3		3	3.4	
	4–6		0	0.0	
	7–10		85	96.6	
Apgar score—5 min	0–3		5	5.7	
	4–6		5	5.7	
	7–10		78	88.6	
Apgar score—10 min	0–3		7	8.0	
	4–6		8	9.0	
	7–10		73	83.0	
NICU	Admission		7	8.0	
	Brief stay		9	10.2	
	No admission		72	81.8	

**Table 2:** Maternal demographic, anthropometric, and tobacco use characteristics. Data are presented as mean ± standard deviation (SD) for continuous variables and as frequency (*n*) and percentage (%) for categorical variables. BMI, body mass index. Tobacco use includes conventional cigarette smoking and use of alternative tobacco or nicotine products. Parity indicates the number of completed pregnancies.

	Minimum		Maximum		Mean	Standard Deviation
Age (years)	18		47		29.88	5.74
Weight (kg)	48		145		81.13	15.23
Weight before pregnancy (kg)	40		138		68.45	15.70
Weight gain (kg)	−18		43		12.67	7.20
BMI at assessment (kg/m^2^)	19.23		51.99		30.31	5.64
Parity	0		6		0.83	0.99
				Frequency (*n*)		Percent (%)
Smoking during pregnancy		Yes		16		18.20
		No		72		81.80
Cigarettes per day during pregnancy		No smoking		72		81.80
		Light		10		11.40
		Moderate		3		3.40
		Heavy		1		1.10
		Smoking alternatives		2		2.30
Smoking only before pregnancy, not during		Yes		21		23.90
		No		67		76.10
Smoking the day prior to delivery		No smoking		67		76.10
		Light		6		6.80
		Moderate		11		12.50
		Heavy		3		3.40
		Smoking alternatives		1		1.10
Alcohol consumption during pregnancy		Yes		1		1.10
		No		87		98.90
Gestational diabetes mellitus		Yes		15		17.00
		No		73		83.00
Diabetes-associated medication		Yes		10		11.40
		No		78		88.60

**Table 3:** Descriptive statistics of umbilical cord blood leptin and visfatin concentrations. Data are presented as median and interquartile range (IQR), with minimum and maximum values.

	Minimum	Maximum	Median	IQR
Leptin (ng/mL)	0.45	6.64	2.20	1.02
Visfatin (ng/mL)	2.90	13.50	6.70	3.48

**Table 4:** Correlations between neonatal anthropometric characteristics and umbilical cord blood leptin and visfatin concentrations. Data are presented as Spearman’s rank correlation coefficients (r), *p*-values, and 95% confidence intervals (CIs). Adipokine concentrations are expressed in ng/mL.

Group	Adipokine	r	*p*	95% CI
Birth weight (kg)	Leptin	−0.009	0.933	−0.224 to 0.207
	Visfatin	0.359	<0.001	0.156 to 0.533
Head circumference (cm)	Leptin	−0.028	0.793	−0.242 to 0.188
	Visfatin	0.233	0.029	0.018 to 0.427
Length (cm)	Leptin	0.092	0.392	−0.126 to 0.302
	Visfatin	0.201	0.061	−0.016 to 0.399

**Table 5:** Umbilical cord blood leptin and visfatin concentrations according to gestational diabetes mellitus status and neonatal weight category. Data are presented as median [IQR] for each group. Overall *p*-values were obtained using the Kruskal–Wallis test for comparisons among three groups or the Mann–Whitney U test for comparisons between two groups. Post hoc *p*-values and 95% confidence intervals (CI) are presented for pairwise comparisons across groups using Hodges–Lehmann estimate. Adipokine concentrations are expressed in ng/mL.

Adipokine	Group	Median (IQR)	*p*	95% CI	Post Hoc *p*		95% CI for Pairwise Differences
Leptin	GDM	2.22 (1.09)	0.002	2.33 to 2.88	-		−1.007 to −0.237
	No GDM	1.50 (0.77)		1.46 to 2.18			
Visfatin	GDM	6.40 (3.50)	0.965	5.29 to 7.70			−1.40 to 1.40
	No GDM	6.80 (3.45)		6.07 to 7.30			
Leptin	SGA	2.17 (1.54)	0.885	1.59 to 3.39	AGA-SGA	0.809	−0.475 to 0.717
	AGA	2.20 (0.96)		2.16 to 2.72	AGA-LGA	0.636	−0.30 to 0.41
	LGA	2.19 (1.25)		2.06 to 2.97	SGA-LGA	0.778	−0.77 to 0.56
Visfatin	SGA	6.00 (2.13)	<0.001	5.33 to 6.84	AGA-SGA	0.766	−1.80 to 1.00
	AGA	6.05 (4.33)		5.05 to 6.36	AGA-LGA	<0.001	−3.70 to −1.20
	LGA	8.25 (3.05)		7.27 to 9.34	SGA-LGA	0.008	−3.50 to −0.90

**Table 6:** Multiple linear regression analysis of the associations between umbilical cord blood leptin and visfatin concentrations and selected maternal and neonatal characteristics, respectively. B, unstandardized regression coefficient; β, standardized regression coefficient; CI, confidence interval; R, multiple correlation coefficient; R^2^, coefficient of determination; adjusted R^2^, adjusted coefficient of determination; GDM, gestational diabetes mellitus. Overall *p*-values refer to the significance of the respective regression models.

Model	Association Variable	B	β	95% CI	*p*	R	R^2^	Adjusted R^2^	Overall *p*
Leptin	Weight fluctuation	0.005	0.120	−0.004 to 0.015	0.260	0.304	0.093	0.071	0.016
	GDM presence	0.29	0.256	0.053 to 0.528	0.017				
Visfatin	Birth weight	0.0003	0.275	−0.00009 to 0.0006	0.143	0.452	0.205	0.166	<0.001
	Head circumference	−0.023	−0.085	−0.094 to 0.048	0.524				
	Length	−0.025	−0.133	−0.080 to 0.029	0.364				
	Weight category	0.165	0.367	0.063 to 0.267	0.002				

## Data Availability

The raw absorbance data and standard curves generated during the ELISA analyses are provided in Supplementary Figure S1. The datasets generated and/or analyzed during the current study are not publicly available due to privacy and ethical restrictions concerning human participant data but are available from the corresponding author upon reasonable request, subject to the applicable ethical and data protection requirements.

## Supplementary Materials

The following supporting information can be downloaded at https://www.mdpi.com/article/10.3390/ijms27198843/s1.

## Abbreviations

The following abbreviations are used in this manuscript:

GDM	Gestational Diabetes Mellitus
LGA	Large for Gestational Age
SGA	Small for Gestational Age
AGA	Appropriate for Gestational Age
FGR	Fetal Growth Restriction
NAMPT	NicotinAMide PhosphoribosylTransferase
BMI	Body Mass Index
NICU	Neonatal Intensive Care Unit
