# Supplementary material for: Exploring the Role of Umbilical Cord Blood Leptin and Visfatin in Fetal Growth: Associations with Gestational Diabetes Mellitus and Neonatal Anthropometric Measurements
Source: Int J Mol Sci. 2026 Oct 3;27(19):8843. doi: 10.3390/ijms27198843 (PMC13665405; doi:10.3390/ijms27198843)
Supplement: Supplementary file 1 [file ijms-27-08843-s001.zip › ijms-4509666-supplementary.pdf]

Supplementary Table S1. Clinical and neonatal characteristics of cases with a worsened Apgar score

Abbreviations: Apgar, Appearance, Pulse, Grimace, Activity, and Respiration score; GDM, gestational diabetes mellitus; NICU, neonatal intensive care unit; RDS, respiratory distress syndrome; SGA, small for gestational age.

Case code	1-min Apgar	5-min Apgar	10-min Apgar	Birth weight (g)	Delivery route	NICU stay	Clinical information
7	7	7	3	2620	Cesarean section	7 days	The newborn developed worsening expiratory grunting accompanied by respiratory distress. SGA; maternal GDM.
20	7	6	3	3380	Vaginal	6 days	The newborn developed worsening expiratory grunting accompanied by respiratory distress and a decline in heart rate.
22	7	7	6	2800	Cesarean section	6 days	The newborn presented with tachycardia and subsequently developed worsening respiratory distress with expiratory grunting, followed by a decline in heart rate.
31	7	7	6	2840	Cesarean section	3 days	The newborn developed hypoglycemia.
32	7	7	6	2475	Cesarean section	3 days	The newborn developed hypoglycemia.
38	7	5	5	3210	Cesarean section	6 days	The newborn developed worsening expiratory grunting accompanied by respiratory distress and a decline in heart rate.
64	7	6	6	2620	Cesarean section	3 days	The newborn developed worsening expiratory grunting accompanied by respiratory distress. SGA.
72	7	3	3	3400	Cesarean section	29 days	The newborn developed bradycardia.
111	7	6	6	3810	Cesarean section	3 days	Reduced respiratory effort and muscle tone.
113	7	7	3	3930	Cesarean section	14 days	Respiratory distress syndrome (RDS).
							Neonatal hypoglycemia

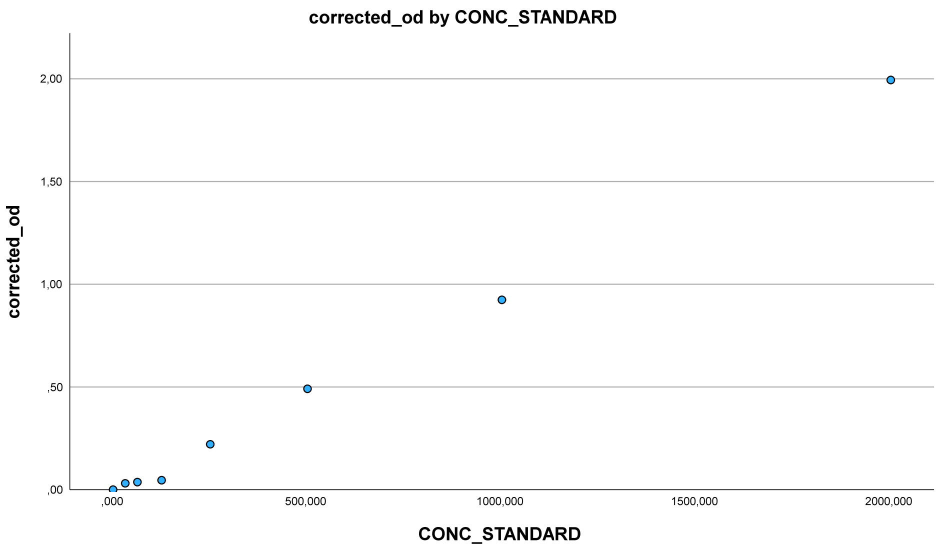

Supplementary Figure S 1. Raw absorbance data and standard curves. Raw absorbance measurements and the corresponding standard curves used for the ELISA analyses are presented.

	1	2	3	4	5	6	7	8	9	10	11	12			
A	0,466	0,967	0,49	0,14	0,22	0,19	0,71	0,177	0,466	0,944	0,451	0,152	OD	duplicate 1	diluted samples 1:4
B	0,222	0,145	0,694	0,202	0,203	0,146	0,246	0,199	0,261	0,512	0,481	0,811			no blank correction
C	0,557	0,701	0,148	0,284	0,196	0,177	0,339	0,649	0,221	0,599	0,399	1,595			
D	0,697	0,404	1,776	0,215	0,009	0,245	1,319	0,638	0,349	0,397	0,249	0,248			
E	0,29	0,355	0,425	1,188	1,024	0,634	0,249	0,499	0,442	0,318	0,489	1,309			
F	0,406	0,413	0,709	0,416	1,244	0,716	1,204	0,713	0,246	0,349	0,261	0,311			
G	0,338	0,581	1,311	0,61	0,455	0,209	1,121	0,891	0,649	0,581	0,364	1,131			
H	0,554	0,274	0,885	0,432	0,681	1,109	1,502	1,167	0,342	0,506	0,495	0,466			
	1	2	3	4	5	6	7	8	9	10	11	12			
A	0,468	0,963	0,45	0,148	0,212	0,082	0,649	0,213	0,468	0,986	0,489	0,136	OD	duplicate 2	
B	0,216	0,127	0,666	0,188	0,203	0,154	0,188	0,189	0,161	0,338	0,375	0,733			
C	0,337	0,543	0,224	0,44	0,168	0,047	0,331	0,579	0,201	0,527	0,327	1,521			
D	0,581	0,546	1,66	0,357	0,017	0,309	1,397	0,584	0,421	0,377	0,207	0,188			
E	0,272	0,367	0,525	0,928	1,148	0,616	0,273	0,283	0,478	0,288	0,447	1,163			
F	0,55	0,553	0,847	0,536	1,316	0,856	1,146	0,859	0,34	0,425	0,311	0,431			
G	0,286	0,587	1,257	0,618	0,481	0,213	1,167	0,815	0,569	0,531	0,41	1,113			
H	0,618	0,182	0,843	0,49	0,591	0,825	1,442	1,105	0,43	0,432	0,455	0,446			
